# The Effect of Psycho-Social Problems on Risky Behaviors in People Living With HIV in Tehran, Iran 

**Published:** 2018-06

**Authors:** Mehrnaz Rasoolinejad, Nasrin Abedinia, Ahmad Ali Noorbala, Minoo Mohraz, Zahra Bayat Jozani, Banafsheh Moradmand Badie

**Affiliations:** 1 Department of Infectious Diseases, Imam Khomeini Hospital, Tehran University of Medical Sciences, Tehran, Iran; 2 Iranian Research Center for HIV/AIDS, Tehran University of Medical Sciences, Tehran, Iran; 3 Iranian Institute for Reduction of High Risk Behaviors, Tehran University of Medical Sciences, Tehran, Iran; 4 Maternal Neonatal & Fetal Health Research Center, Tehran University of Medical Sciences, Tehran, Iran; 5 Department of Psychiatric, Psychosomatic Ward, Tehran University of Medical Sciences, Tehran, Iran; 6 Manager of Tehran Positive Club, Iranian Institute for Reduction of High Risk Behaviors, Tehran University of Medical Sciences , Tehran, Iran; 7 Department of Public Health, Faculty of Public Health, Flinders University, Flinders, Australia

**Keywords:** Stigma, Psychiatric Disorders, Coping Mechanisms, Risky Behaviors, Human Immunodeficiency Virus / Acquired Immune Deficiency Syndrome (HIV/AIDS)

## Abstract

**Objective:** Over the past years, the prevalence and the progression rate of HIV infection in Iran especially through high-risk sexual relationships have regrettably been reported at very high levels. This cross-sectional study tries to analyze stigma, mental health, and coping skills on risky behaviors in HIV-positive adults in Tehran- Iran.

**Materials and methods:** This cross-sectional study was conducted on a sample of 450 HIV-positive adults. Participants completed a socio-demographic questionnaire, the General HealthQuestionnaire-28, the Berger HIV Stigma Scale as well as the Lazarus Ways of Coping Questionnaire (WOCQ). To analyze the data, the independent-samples t-test and Pearson Correlation were used.

**Results:** The findings of this study revealed that mental health, stigma, and avoidance-escape coping mechanisms were correlated with risky behaviors (p ˂ 0.05).Furthermore, the amount of stigma among female individuals compared to men was reported at higher levels and mental health status in the given group was lower than among male individuals.

**Conclusion:** It seems that psychological treatment techniques could be effective in improving mental health and reducing risky behaviors.

## Introduction

According to the World Health Organization (WHO), acquired immune deficiency syndrome (AIDS) kills many people each year and presently over 42 million people worldwide have been diagnosed with the infection. Statistics released by Iran’s Ministry of Health and Medical Education also indicate that the annual growth rate of the HIV infection and transmission is 10% (1). People suffering from HIV/AIDS are affected by numerous social problems, the abuse of dominant social attitudes, and stigma, especially social stigma. Mental disorders and emotional complexities of HIV/AIDS are almost universal and more than half of patients demonstrate diagnosable emotional abnormalities and mental disorders such as depression, manic depression, psychosis, anxiety, substance abuse, and suicide attempts (2, 3). Our aim in this study was to identify the most important psychological variables in order to advance comprehensive treatment strategies, increase treatment effectiveness, and contribute specifically to the issue of prevention.

## Materials and methods

The present study was a cross sectional study lasting for a year with intervals from 2015 to 2016 in the Infectious Diseases and Behavioral Health Clinic at Imam Khomeini Hospital. Among all patients with HIV/AIDS whose infections had been confirmed by two positive tests (Elisa HIV-ab and Western-Blot HIV-ab), 450 HIV-positive individuals were selected using the convenience sampling method (all HIV positive patients from the clinic of Imam Khomeini Hospital) and were included in the study following explanations giving information about the study as well as obtaining informed consent. The inclusion criteria in this study were all patients with reading and writing ability receiving anti-retroviral treatment and willing to participate in the study. The participants of this study were granted financial rewards after completing the questionnaires. The data collection instruments in this study included a demographic characteristics questionnaire, the Berger HIV Stigma Scale, General Health Questionnaire-28, and the Lazarus Ways of Coping Questionnaire (WOCQ).

The Berger HIV Stigma Scale examines psychological aspects of stigma in patients affected with HIV. This questionnaire is associated with depression and quality of life (self-esteem, social support and conflicts) and includes 4 sub-scales of personal stigma for experiences with rejection, loss of job, discrimination, and disruption of social relationships. Lack of disclosure is also related to fear and anxiety caused by the disease. Negative self-image is the fear of stigma and people’s reactions as well as sense of guilt. ‘Public attitudes’ refers to people’s attitudes towards HIV-infected individuals in a way that such patients assume themselves abhorrent and dirty. The given scale consists of 40 items scored based on a Likert-type scale and scores for each item are from 1 to 4 with a range between 40 and 160. The validity and reliability coefficients for the given questionnaire have been reported at0.83 and 0.81, respectively (3).

The 28-item General Health Questionnaire (GHQ-28) is comprised of four sub-scales and each scale includes 7 items. The given scales are the somatic symptoms scale, including issues about how people feel about their health status and their fatigue accompanied by physical symptoms; the scale of symptoms of anxiety and sleep disorders which are associated with insomnia and anxiety; the social functioning scale which measures the ability of individuals to deal with demands and problems of everyday life; and the scale of symptoms of depression which includes chronic depression and suicidal tendencies. There is a score for each sub-scale and a score is also assigned to the total score obtained by respondents. The internal consistency of the GHQ-28 using Cranach's alpha method was 0.87. The reliability coefficient of the sub-scales was between 0.50 and 0.81 and its sensitivity and specificity values were reported equal to 0.86 and 0.82, respectively. The GHQ-28 scoring method is designed so that options A to D are given scores 0, 1, 2, and 3; respectively. As a result, each individual’s score for each sub-scale is from 0 to 21 and in total from 0 to 84. The scores of each scale are also calculated separately; then, the scores of scales are summed up, and the total score is obtained in the end (4).

The Lazarus Ways of Coping Questionnaire (WOCQ) is based on Lazarus and Folkman's Transactional Model of Stress and Coping. This 66-item questionnaire evaluates a wide range of thoughts and actions of individuals encountering internal or external stressful situations. It should be noted that there are two general coping strategies, problem-focused coping style and emotion-focused coping style, employed by individuals who are facing problems. In this respect, individuals make use of problem-focused coping style when they feel that they can do something about a problem, while they resort to emotion-focused coping style if they assume that the situation goes beyond their abilities. However, individuals often utilize a combination of both techniques which brings about a more reliable outcome. This test is also comprised of 8 sub-scales: 4 sub-scales are associated with emotion-focused coping style including confrontation, avoidance, self-control, and distancing coping strategies and 4 sub-scales are related to problem-focused coping style such as seeking social support, accepting responsibility, planned problem-solving, and positive reappraisal. The scoring method is also based on a four-point Likert-type scale (0= Does not apply or not used, (0 = Does not apply or not used, 1 = used a bit, 2 = used to some extent and 3 = used a great deal) 1 = Used quite a bit, 2 = Used to some extent, and 3 = Used a great deal). In a study, the reliability of the questionnaire using the internal consistency method (Cranach's alpha coefficient) was estimated at0.80 (5). To investigate risky behavior, a researcher-designed questionnaire comprised of 18 items was used which included the following issues: sex with multiple partners, unprotected sex, use of shared syringes, consumption of alcohol and psychoactive drugs in situations like parties and so on.

SPSS software (Version 20) was used for data analysis (SPSS, Inc., Chicago, Il, USA). The data obtained were analyzed using descriptive statistics (mean, standard deviation, frequency, and percentage) as well as inferential statistics, including the independent t-test, analysis of variance (ANOVA) and the Pearson correlation coefficient test. The level of significance in this study was 95%.

## Results

A total number of 450 HIV-positive patients participated in this study. The mean and the standard deviation of age in the group of women was 38.52±7.86 and among male individuals was38.31±8.53. In terms of marital status, 34.8% of women (64 individuals) and 26.7% of men (71 patients) were married. As well, 57.2% (91 individuals) and 50.2% (106 individuals) of women and men respectively had the middle birth order. In addition, 15.5% (28 patients) of women and 9.1% of men (24 individuals) were homeowners. A history of referrals to psychiatrists was seen in5.6% of women (99 patients) and 45.6% of men (119 patients); furthermore, 46.2% of women (79 patients) and 40.8% of men (102 patients) had a history of psychiatric illnesses. In terms of taking psychiatric drugs, 54.4% of women (96 patients) and 48.6% of men (125 individuals) were using such medications. Family history of psychiatric disorders was seen in 15.3% of women (18 individuals) and 12.5% of men (19 patients). In addition, a history of physical and sexual disorders in the group of women was reported at 23.7% (40 patients) and 40.9% (38 patients), respectively. Such values among male patients were 28.2% (73 individuals) for physical illnesses and 13% (19 patients) for sexual ones. A history of suicide attempts was also reported among 36.7% of women (61 patients) and 33.3% of men (83 patients). Stress was suffered by94.6% of women (174 patients) and 97% of men (255 patients) and 4.3% of women (8 patients) 26.7% of men (71 patients) were also inflicted with hepatitis. The mean and standard deviation of CD4 in the group of women was 527.64 ± 213.72 and among male patients was 641.63 ± 192.41. In terms of the cases of infection among women by way of their husbands, they stated that they did not know how their husbands had been inflicted with HIV/AIDS. The demographic characteristics of participants in the present study are shown in Table 1.

**Table1:** Demographic characteristics of HIV-positive patients in this study samples

**Variables**		**n (%)**
Gender		
Female		184 (40.9%)
Male		266 (59.1%)
Age		
37 ˃		253 (56.2%)
37 ˂		197 (43.8%)
37.29 ± 8.34		
Marital status		
Single		206 (45.8%)
Married		135 (30%)
Divorced		55 (12.2%)
Widowed		45 (10%)
Temporarily married		3 (0.7%)
Others		6 (1.3%)
Having children		154 (61.1%)
Child birth ranking in family		
First		93 (25.1%)
Middle		197 (53.2%)
Last		80 (21.6%)
History of referral to psychiatrist		218 (49.7%)
History of mental disorders		181 (43%)
History of using psychiatric medications		221 (51%)
History of psychiatric disorders in family		37 (13.7%)
Suicidal attempts		144 (34.7%)
Education in women		
Under diploma		130 (49.8%)
Diploma		93 (35.6%)
Upper diploma		38 (14.5%)
Education in men		
Under diploma		197 (57.6%)
Diploma		99 (28.9%)
Upper diploma		46 (13.5%)
Job status		
Housewife		73 (20.3%)
Unemployed		66 (18.3%)
Worker		18 (5%)
Employed		50 (13.9%)
Self-employed		143 (39.7%)
Driver		10 (2.8%)
Ways of disease infection		
By spouse		110 (24.4%)
Through injection and addiction		185 (41.1%)
Via contaminated blood products		18 (4%)
By sexual relationships		131 (29.1%)
Through dentistry		2 (0.4%)
Via tattoos		4 (0.8%)
Housing status		
Homeowner		52 (11.7%)
Rental housing		205 (46.1%)
Living with family		69 (38%)
Other cases (dormitory and so on)		19 (4.3%)
Hepatitis		79 (17.6%)
Number of CD4	487.81 ± 203.47	

**Table 2:** Stigma scores among HIV-positive patients base on gender

**Variables**	**Female** **M ± SD**	**Male** **M ± SD**	**Total Score** **M ± SD**	**p**
**Stigma**				
Personal stigma	47.69 ± 10.45	46.73 ± 7.81	47.12 ± 8.98	0.0001
Disclosure	27.44 ± 4.95	26.79 ± 4.70	27.06 ± 4.81	0.315
Negative image	19.99 ± 5.90	20.60 ± 4.73	20.35 ± 5.24	0.001
Public attitude	21.54 ± 4.49	20.49 ± 3.71	20.92 ± 4.07	0.0001
Stigma score	116.67 ± 22.39	114.60 ± 17.99	115.45 ± 19.91	0.001

* M ± SD: Mean & Standard Deviation

As illustrated in Table 3, women received higher scores on the GHQ-28 compared with men which meant that mental health status among female individuals was lower than among men and the difference was statistically significant with a confidence interval of p = 0.046. Furthermore, all the sub-scales of general health except anxiety/insomnia and social function in both groups of men and women were significantly different (p < 0.005). The highest and the lowest disorder prevalence in both groups were reported in the sub-scales of somatization and depression, respectively. According to the Table 3, the scores of patients obtained from GHQ-28 and all its sub-scales were also higher than the cut-off score specified in this questionnaire (Table 3).

According to the data shown in Table 4, men obtained higher scores on Lazarus WOCQ in terms of emotion-focused and problem-focused coping styles compared with women. It seemed that scores on emotion-focused coping style were higher compared with those of the problem-focused coping style but these differences were not significant (Table 4).

As presented in Table 5, the results of this study concerning the relationship between mental health and risky behaviors suggested that mental health (other than somatization) was significantly correlated with high-risk behaviors (p < 0.05). In other words, risky behaviors had a descending trend when mental health status was at higher levels; conversely, risky behaviors increased when mental health had reduced. Furthermore, the results associated with the relationship between stigma and risky behaviors revealed that stigma was significantly correlated with high-risk behaviors, i.e. as stigma increased, risky behaviors had a rising trend; in contrast, high-risk behaviors declined when the amount of stigma had a decreasing trend (p < 0.05). Considering the relationship between coping mechanisms and high-risk behaviors, the findings indicated a significantly negative correlation only between avoidance-escape coping mechanisms and risky behaviors; in other words, the increased use of avoidance-escape coping mechanisms lead to a reduction in risky behaviors; and in reverse, lowered use of avoidance-escape coping mechanisms increased such behaviors (p < 0.01).

## Discussion

The prevalence rate of AIDS in Iran is increasing through sexual intercourse, use of contaminated syringes by injection drug users and, in limited cases, mother-to-child virus transmission. According to reports released by the WHO, the growth rate of the AIDS epidemic is on a worrying rising trend (6).

Thus, the purpose of the present study was to examine the impact of mental health, stigma, and coping mechanisms on risky behaviors among HIV-positive patients. In this respect, results showed that mental health, stigma, and avoidance-escape coping mechanisms were correlated with risky behaviors. Furthermore, the amount of stigma among women was higher but the mental health status in this group was lower than among men.

**Table 3:** General health scores among HIV-positive patients base on gender

**Variables**	**Female** **M ± SD**	**Male** **M ± SD**	**Total Score** **M ± SD**	**p**
**General health**				
Somatization	10.01 ± 4.94	8.74 ± 4.52	9.26 ± 4.73	0.028
Anxiety and insomnia	9.48 ± 5.08	9.35 ± 4.77	9.40 ± 4.89	0.554
Social function	9.33 ± 3.68	9.35 ± 3.89	9.34 ± 3.80	0.335
Depression	7.50 ± 6.06	7.59 ± 5.22	7.55 ± 5.57	0.001
General health	36.33 ± 18.10	35.03 ± 16.50	35.56 ± 17.17	0.046

* M ± SD: Mean & Standard Deviation

**Table 4:** Scores of Lazarus’s coping mechanisms among HIV-positive patients base on gender

**Coping Mechanism**	**Female** **M ± SD**	**Male** **M ± SD**	**Total Score** **M ± SD**	**p**
Confrontation or coping	5.22 ± 3.03	6.22 ± 3.04	5.81 ± 3.07	0.704
Distancing	6.15 ± 3.52	6.70±3.09	6.47 ± 3.28	0.126
Self-control	7.23 ± 4.05	8.07 ± 3.72	7.72 ± 3.88	0.057
Social support	6.85 ± 3.77	6.50 ± 3.24	6.64 ± 3.47	0.069
Responsibility	3.93 ± 2.47	5.21 ± 2.39	4.67 ± 2.50	0.206
Avoidance and escape	7.53 ± 4.10	9.10 ± 43.38	8.46 ± 4.34	0.853
Planned problem-solving	5.66 ± 3.34	6.59 ± 3.38	6.31 ± 3.39	0.733
Positive reappraisal	7.78 ± 4.21	8.47 ± 4.01	8.19 ± 4.10	0.499
Lazarus	50.16 ± 23.67	56.86 ± 23.46	54.12 ± 23.75	0.596
Problem-focused	24.22 ± 12.21	26.76 ± 11.55	25.72 ± 11.88	0.587
Emotion-focused	26.08 ± 12.43	30.20 ± 12.45	28.51 ± 12.59	0.631

* M ± SD: Mean & Standard Deviation

In this regard, the results of a study on 493 homosexual men (MSM: men who have sex with men) in China showed that stigma, anxiety, depression, and avoidance-escape coping mechanisms had effects on attempts to show high-risk behaviors (7). In another report, the findings suggested that HIV-related stressors and internalized stigma were associated with mental health and sexual behaviors. As well, emotional problems could be an intervening factor affecting internalized stigma that somehow influenced anxiety and sexual obsession/hypersexuality. The internalized stigma is also a favorable predictor for unprotected sex among individuals infected with HIV (8, 9). Moreover; Wagner et al. (2012) reported that depression as an important factor affecting condom use over time had an impact on individuals undertaking 7 ART therapies. It was argued that healthcare and ART treatments could increase sexual activities and condom use, but depression could undermine the use and continuation of the ART therapy (10). Considering the relationship between mental health and high-risk behaviors, the results of a similar study also showed that HIV-related stigma was accompanied by symptoms such as depression (β = 0.16, p < 0.001), horror (p = 0.01, β = 0.11), and anxiety (β = 0.05, p = 0.05). In addition, HIV-related stigma was correlated with risky behaviors such as unprotected anal sex, negative HIV serology, or unknown sexual partners (β = 0.06, p = 0.047). The experience of HIV related stigma could also increase the possibility of high-risk behaviors associated with sexual transmission as well as mental and psychological problems. Mental health problems (depression), alcohol consumption, and violent behaviors are similarly associated with risky behaviors among HIV patients (11, 12).

**Table 5:** Relationship between mental health, coping mechanisms, and stigma among HIV-positive patients with high-risk behaviors

**Variables**	**Risky behaviors r (p)**	**Variables**	**Risky behaviors r (p)**
Somatization	0.061 (0.155)	Confrontation or coping	0.053 (0.221)
Anxiety/insomnia	0.100 (0.019)	Distancing	0.063 (0.140)
Social function	0.163 (0.0001)	Self-control	0.004 (0.925)
Depression	0.103 (0.015)	Social function	-0.062 (0.145)
Mental health	0.120 (0.004)	Responsibility	0.055 (0.206)
Personal stigma	0.124 (0.003)	Avoidance and escape	0.110 (0.010)
Disclosure	0.085 (0.044)	Problem-solving	0.012 (0.778)
Negative image	0.098 (0.021)	Positive reappraisal	0.024 (0.568)
Public attitude	0.106 (0.013)	Lazarus	0.046 (0.263)
Stigma	0.129 (0.002)	Problem-focused	0.011 (0.795)
		Emotion-focused	0.074 (0.075)

* r(p) Pearson correlation (confidence level)

In another study conducted in Ghana in this regard, the results suggested that the frequency of using condoms for oral sex was less reported among young men with higher levels of awareness concerning sexually transmitted diseases as well as aging men with higher amounts of stigma. Furthermore, the stigma associated with the use of condoms for anal or vaginal sex was not correlated with different age groups (13). In this respect, Shuper et al. (2014) reported that non-use of condoms among HIV-positive men was due to motivational barriers, lack of awareness, negative attitudes to condoms, as well as having symptoms of depression (14).

According to the results of this study and a review of the related literature in this respect, it was concluded that patients’ mental health status and amount of stigma could have an impact on high-risk behaviors such as unprotected sex, non-use of condoms, and alcohol consumption. Also, the role of psychological factors in risky behaviors was of utmost importance. Given the use of avoidance-escape coping mechanisms that originated from social stigma, it seems that psychological interventions could lead to reduced use of avoidance mechanisms, improvements in mental health, and distancing from social isolation, which in turn could lower the likelihood of risky behaviors.

## Conclusion

Patients’ mental health status and amount of stigma have impact on high-risk behaviors such as unprotected sex, non-use of condoms, and alcohol consumption. The role of psychological factors in risky behaviors was of utmost importance. Psychological interventions lead to reduced use of avoidance mechanisms, improvements in mental health and distancing from social isolation which in turn lowers the likelihood of risky behaviors.
